# Bis(1*H*-imidazole-κ*N* ^3^)bis­(1-naph­tha­lene­acetato-κ^2^ *O*,*O*′)cadmium(II)

**DOI:** 10.1107/S1600536808009409

**Published:** 2008-04-10

**Authors:** Wen-Dong Song, Li-Li Ji, Hong-Mian Wu

**Affiliations:** a College of Science, Guang Dong Ocean University, Zhanjiang 524088, People’s Republic of China; b College of Food Science and Technology, Guang Dong Ocean University, Zhanjiang 524088, People’s Republic of China

## Abstract

In the mononuclear title compound, [Cd(C_12_H_9_O_2_)_2_(C_3_H_4_N_2_)_2_], the Cd^II^ centre has a distorted octa­hedral coordination geometry defined by four O atoms from two naphthalene­acetate ligands and two N atoms from two imidazole ligands. The mol­ecules are linked by N—H⋯O hydrogen bonds, forming a layer network.

## Related literature

For related literature, see: Duan *et al.* (2007 ▶); Liu *et al.* (2006 ▶).
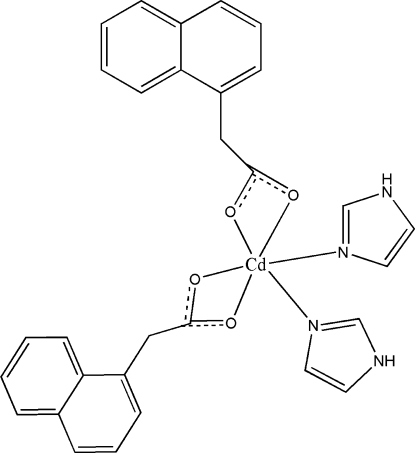


## Experimental

### 

#### Crystal data

[Cd(C_12_H_9_O_2_)_2_(C_3_H_4_N_2_)_2_]
*M* *_r_* = 618.95
Monoclinic, 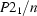

*a* = 8.5275 (3) Å
*b* = 17.1596 (7) Å
*c* = 19.1198 (6) Å
β = 100.735 (2)°
*V* = 2748.81 (17) Å^3^
*Z* = 4
Mo *K*α radiation
μ = 0.84 mm^−1^
*T* = 296 (2) K
0.26 × 0.23 × 0.21 mm

#### Data collection

Bruker APEXII area-detector diffractometer
Absorption correction: multi-scan (*SADABS*; Sheldrick, 1996 ▶) *T*_min_ = 0.812, *T*_max_ = 0.844
26061 measured reflections
5399 independent reflections
3336 reflections with *I* > 2σ(*I*)
*R*_int_ = 0.089

#### Refinement

*R*[*F* ^2^ > 2σ(*F* ^2^)] = 0.051
*wR*(*F* ^2^) = 0.142
*S* = 0.99
5399 reflections
340 parameters
H-atom parameters constrained
Δρ_max_ = 0.57 e Å^−3^
Δρ_min_ = −1.16 e Å^−3^

### 

Data collection: *APEX2* (Bruker, 2004 ▶); cell refinement: *SAINT* (Bruker, 2004 ▶); data reduction: *SAINT*; program(s) used to solve structure: *SHELXS97* (Sheldrick, 2008 ▶); program(s) used to refine structure: *SHELXL97* (Sheldrick, 2008 ▶); molecular graphics: *XP* in *SHELXTL* (Sheldrick, 2008 ▶); software used to prepare material for publication: *SHELXTL*.

## Supplementary Material

Crystal structure: contains datablocks I, global. DOI: 10.1107/S1600536808009409/ng2436sup1.cif

Structure factors: contains datablocks I. DOI: 10.1107/S1600536808009409/ng2436Isup2.hkl

Additional supplementary materials:  crystallographic information; 3D view; checkCIF report

## Figures and Tables

**Table 1:** Hydrogen-bond geometry (Å, °)

*D*—H⋯*A*	*D*—H	H⋯*A*	*D*⋯*A*	*D*—H⋯*A*
N2—H2⋯O4^i^	0.86	1.92	2.735 (6)	159
N4—H4*A*⋯O2^ii^	0.86	1.95	2.772 (6)	159

Symmetry codes: (i) 
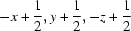
; (ii) 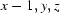.

**Fig. 1.**
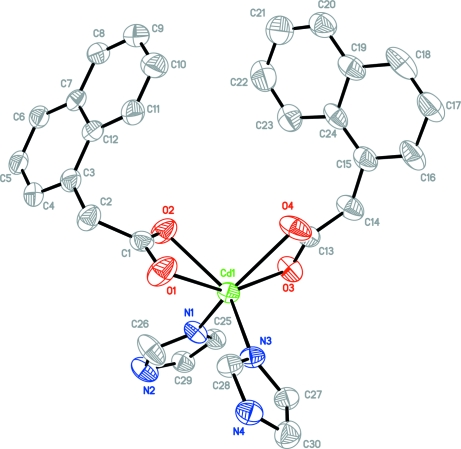
The structure of (I), showing the atomic numbering scheme. Non-H atoms are shown as 30% probability displacement ellipsoids.

**Fig. 2.**
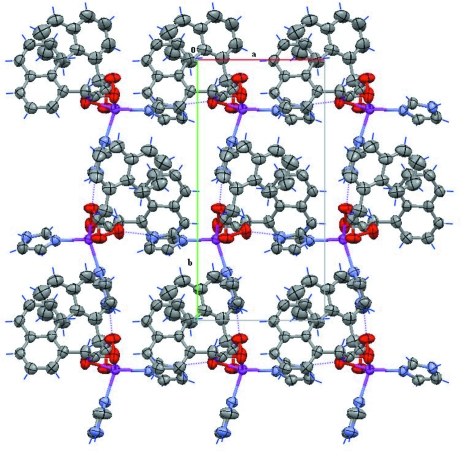
The packing of structure (I).

## supplementary crystallographic information

### Comment

naphthaleneacetate is nice ligand which has versatile bonding modes to metal ions and easily forms simple complexes (Liu *et al.*, 2006; Duan *et al.*, 2007). Recently, we obtained the mononuclear cadmium complex, (I), its crystal structure is reported here. Recently, we obtained the title novel mononuclear complex (I), by the reaction of cadmium chloride, 1-naphthylacetic acid and imidazole in an aqueous solution, and its crystal structure is reported here.

As depicted in Fig. 1, the Cd^II^ centre in (I) presents a distorted octahedral coordination geometry, defined by four O atoms from two 1-naphthaleneacetate ligands, and two N atoms from two imidazole ligands. The structural packing is governed by N—H···o hydrogen bonding interaction (Table 1) to form a layered network (Fig. 2).

### Experimental

The title complex was prepared by the addition of a stoichiometric amount of cadmium chloride (1 mmol) and imidazole (1 mmol) to a hot aqueous solution(10 ml) of 1-naphthylacetic acid (1 mmol). the pH was then adjusted to 7.0 to 8.0 with NaOH (1 mmol). The resulting solution was filtered, and colorlee single crystals were obtained at room temperature over several days.

### Refinement

All H-atoms were placed in calculated positions with C—H = 0.93–0.97 Å, N—H = 0.86 Å; refined using a riding model with *U*_iso_(H) = 1.2 or 1.5 *U*_eq_(C, N).

### Crystal data

**Table:**

[Cd(C_12_H_9_O_2_)_2_(C_3_H_4_N_2_)_2_]	*F*_000_ = 1256
*M**_r_* = 618.95	*D*_x_ = 1.496 Mg m^−^^3^
Monoclinic, *P*2_1_/*n*	Mo *K*α radiation λ = 0.71073 Å
Hall symbol: -P 2yn	Cell parameters from 5300 reflections
*a* = 8.5275 (3) Å	θ = 1.3–28.0º
*b* = 17.1596 (7) Å	µ = 0.84 mm^−^^1^
*c* = 19.1198 (6) Å	*T* = 296 (2) K
β = 100.735 (2)º	Block, colorless
*V* = 2748.81 (17) Å^3^	0.26 × 0.23 × 0.21 mm
*Z* = 4	

### Data collection

**Table:**

Bruker APEXII area-detector diffractometer	5399 independent reflections
Radiation source: fine-focus sealed tube	3336 reflections with *I* > 2σ(*I*)
Monochromator: graphite	*R*_int_ = 0.089
*T* = 296(2) K	θ_max_ = 26.0º
φ and ω scans	θ_min_ = 2.2º
Absorption correction: multi-scan(SADABS; Sheldrick, 1996)	*h* = −9→10
*T*_min_ = 0.812, *T*_max_ = 0.844	*k* = −16→21
26061 measured reflections	*l* = −23→23

### Refinement

**Table:**

Refinement on *F*^2^	Secondary atom site location: difference Fourier map
Least-squares matrix: full	Hydrogen site location: inferred from neighbouring sites
*R*[*F*^2^ > 2σ(*F*^2^)] = 0.051	H-atom parameters constrained
*wR*(*F*^2^) = 0.143	*w* = 1/[σ^2^(*F*_o_^2^) + (0.0533*P*)^2^ + 1.0058*P*] where *P* = (*F*_o_^2^ + 2*F*_c_^2^)/3
*S* = 0.99	(Δ/σ)_max_ = 0.001
5399 reflections	Δρ_max_ = 0.57 e Å^−^^3^
340 parameters	Δρ_min_ = −1.16 e Å^−^^3^
Primary atom site location: structure-invariant direct methods	Extinction correction: none

### Special details

**Table:**

Geometry. All e.s.d.'s (except the e.s.d. in the dihedral angle between two l.s. planes) are estimated using the full covariance matrix. The cell e.s.d.'s are taken into account individually in the estimation of e.s.d.'s in distances, angles and torsion angles; correlations between e.s.d.'s in cell parameters are only used when they are defined by crystal symmetry. An approximate (isotropic) treatment of cell e.s.d.'s is used for estimating e.s.d.'s involving l.s. planes.
Refinement. Refinement of *F*^2^ against ALL reflections. The weighted *R*-factor *wR* and goodness of fit *S* are based on *F*^2^, conventional *R*-factors *R* are based on *F*, with *F* set to zero for negative *F*^2^. The threshold expression of *F*^2^ > σ(*F*^2^) is used only for calculating *R*-factors(gt) *etc*. and is not relevant to the choice of reflections for refinement. *R*-factors based on *F*^2^ are statistically about twice as large as those based on *F*, and *R*- factors based on ALL data will be even larger.

### Fractional atomic coordinates and isotropic or equivalent isotropic displacement parameters (Å^2^)

**Table:**

	*x*	*y*	*z*	*U*_iso_*/*U*_eq_	
C1	0.3023 (7)	0.6405 (3)	0.3585 (3)	0.0583 (14)	
C2	0.3810 (7)	0.6218 (4)	0.4342 (3)	0.0694 (16)	
H2A	0.3450	0.6594	0.4656	0.083*	
H2B	0.3449	0.5708	0.4463	0.083*	
C3	0.5602 (7)	0.6219 (3)	0.4484 (2)	0.0581 (14)	
C4	0.6434 (8)	0.6822 (3)	0.4844 (3)	0.0656 (16)	
H4	0.5878	0.7224	0.5016	0.079*	
C5	0.8115 (8)	0.6849 (3)	0.4962 (3)	0.0639 (15)	
H5	0.8652	0.7265	0.5212	0.077*	
C6	0.8959 (7)	0.6271 (3)	0.4713 (2)	0.0595 (14)	
H6	1.0067	0.6297	0.4790	0.071*	
C7	0.8159 (7)	0.5633 (3)	0.4339 (2)	0.0540 (13)	
C8	0.9015 (7)	0.5033 (4)	0.4069 (3)	0.0649 (15)	
H8	1.0124	0.5052	0.4145	0.078*	
C9	0.8232 (9)	0.4431 (4)	0.3701 (3)	0.0785 (18)	
H9	0.8802	0.4038	0.3525	0.094*	
C10	0.6560 (9)	0.4402 (4)	0.3585 (4)	0.088 (2)	
H10	0.6025	0.3991	0.3326	0.105*	
C11	0.5716 (7)	0.4966 (4)	0.3845 (3)	0.0716 (16)	
H11	0.4609	0.4927	0.3771	0.086*	
C12	0.6464 (6)	0.5606 (3)	0.4223 (2)	0.0543 (13)	
C13	0.2197 (7)	0.6142 (3)	0.1108 (2)	0.0593 (14)	
C14	0.2727 (9)	0.5800 (4)	0.0458 (3)	0.0794 (19)	
H14A	0.1911	0.5918	0.0046	0.095*	
H14B	0.3691	0.6070	0.0394	0.095*	
C15	0.3051 (9)	0.4945 (4)	0.0457 (3)	0.0718 (17)	
C16	0.2010 (9)	0.4454 (4)	0.0041 (3)	0.092 (2)	
H16	0.1081	0.4658	−0.0230	0.111*	
C17	0.2301 (11)	0.3654 (5)	0.0011 (4)	0.101 (3)	
H17	0.1559	0.3335	−0.0272	0.121*	
C18	0.3651 (12)	0.3339 (5)	0.0390 (5)	0.102 (3)	
H18	0.3832	0.2806	0.0361	0.122*	
C19	0.4786 (9)	0.3811 (4)	0.0827 (3)	0.0738 (18)	
C20	0.6198 (11)	0.3493 (5)	0.1221 (4)	0.091 (2)	
H20	0.6394	0.2962	0.1192	0.109*	
C21	0.7265 (12)	0.3946 (6)	0.1637 (4)	0.113 (3)	
H21	0.8192	0.3730	0.1898	0.135*	
C22	0.6983 (11)	0.4739 (6)	0.1678 (4)	0.108 (3)	
H22	0.7731	0.5048	0.1968	0.129*	
C23	0.5650 (10)	0.5074 (4)	0.1308 (3)	0.085 (2)	
H23	0.5502	0.5608	0.1348	0.102*	
C24	0.4467 (9)	0.4625 (3)	0.0858 (3)	0.0684 (18)	
C25	−0.1907 (7)	0.6672 (3)	0.2707 (3)	0.0589 (14)	
H25	−0.1394	0.6407	0.3110	0.071*	
C26	−0.2362 (7)	0.7286 (3)	0.1729 (3)	0.0626 (15)	
H26	−0.2210	0.7536	0.1315	0.075*	
C27	−0.3772 (8)	0.7215 (4)	0.1936 (3)	0.0734 (16)	
H27	−0.4758	0.7394	0.1699	0.088*	
C28	0.2489 (10)	0.8574 (4)	0.2921 (3)	0.099 (3)	
H28	0.2326	0.8398	0.3362	0.118*	
C29	0.2722 (7)	0.8611 (3)	0.1845 (3)	0.0635 (15)	
H29	0.2723	0.8466	0.1376	0.076*	
C30	0.3157 (7)	0.9321 (4)	0.2124 (3)	0.0698 (16)	
H30	0.3521	0.9743	0.1894	0.084*	
Cd1	0.14721 (4)	0.68863 (2)	0.227269 (18)	0.05146 (17)	
N1	0.2286 (6)	0.8142 (2)	0.2344 (2)	0.0604 (12)	
N2	0.2951 (7)	0.9287 (3)	0.2801 (3)	0.0800 (15)	
H2	0.3095	0.9664	0.3103	0.096*	
N3	−0.1180 (5)	0.6943 (2)	0.2210 (2)	0.0542 (11)	
N4	−0.3455 (6)	0.6822 (3)	0.2569 (3)	0.0648 (13)	
H4A	−0.4137	0.6697	0.2829	0.078*	
O1	0.1546 (5)	0.6372 (3)	0.3436 (2)	0.0823 (9)	
O2	0.3806 (5)	0.6581 (3)	0.3125 (2)	0.0823 (9)	
O3	0.2221 (6)	0.6855 (2)	0.1175 (2)	0.0803 (9)	
O4	0.1694 (6)	0.5712 (2)	0.1548 (2)	0.0803 (9)	

### Atomic displacement parameters (Å^2^)

**Table:**

	*U*^11^	*U*^22^	*U*^33^	*U*^12^	*U*^13^	*U*^23^
C1	0.051 (4)	0.061 (4)	0.066 (3)	0.015 (3)	0.019 (3)	0.004 (3)
C2	0.059 (4)	0.093 (5)	0.059 (3)	0.003 (3)	0.016 (3)	0.003 (3)
C3	0.056 (4)	0.074 (4)	0.044 (3)	0.010 (3)	0.009 (3)	0.005 (3)
C4	0.073 (5)	0.074 (4)	0.048 (3)	0.014 (3)	0.006 (3)	−0.005 (3)
C5	0.076 (5)	0.064 (4)	0.048 (3)	−0.007 (3)	0.001 (3)	0.000 (3)
C6	0.054 (4)	0.064 (4)	0.057 (3)	−0.002 (3)	0.001 (3)	0.007 (3)
C7	0.056 (4)	0.062 (3)	0.043 (2)	0.004 (3)	0.005 (2)	0.012 (2)
C8	0.054 (4)	0.070 (4)	0.068 (3)	0.011 (3)	0.004 (3)	0.002 (3)
C9	0.076 (5)	0.069 (4)	0.089 (4)	0.020 (4)	0.012 (4)	−0.002 (3)
C10	0.088 (6)	0.067 (4)	0.101 (5)	0.003 (4)	0.002 (4)	−0.019 (4)
C11	0.054 (4)	0.075 (4)	0.081 (4)	−0.005 (3)	−0.001 (3)	−0.004 (3)
C12	0.050 (3)	0.061 (3)	0.051 (3)	0.002 (3)	0.006 (3)	0.005 (2)
C13	0.070 (4)	0.060 (4)	0.050 (3)	0.011 (3)	0.017 (3)	0.009 (2)
C14	0.109 (6)	0.077 (4)	0.061 (3)	0.011 (4)	0.036 (4)	0.007 (3)
C15	0.092 (5)	0.078 (4)	0.053 (3)	0.004 (4)	0.034 (3)	−0.011 (3)
C16	0.095 (6)	0.103 (6)	0.084 (4)	0.011 (5)	0.030 (4)	−0.037 (4)
C17	0.092 (6)	0.097 (6)	0.121 (6)	−0.013 (5)	0.038 (5)	−0.055 (5)
C18	0.118 (7)	0.078 (5)	0.130 (6)	−0.013 (5)	0.076 (6)	−0.036 (5)
C19	0.098 (6)	0.061 (4)	0.072 (4)	0.002 (4)	0.041 (4)	−0.009 (3)
C20	0.127 (7)	0.072 (5)	0.081 (4)	0.014 (5)	0.040 (5)	0.002 (4)
C21	0.145 (9)	0.113 (7)	0.080 (5)	0.028 (6)	0.019 (5)	0.005 (5)
C22	0.122 (8)	0.106 (7)	0.091 (5)	−0.014 (6)	0.007 (5)	−0.020 (5)
C23	0.120 (7)	0.065 (4)	0.074 (4)	−0.007 (5)	0.027 (4)	−0.002 (4)
C24	0.104 (5)	0.057 (4)	0.055 (3)	−0.010 (4)	0.043 (4)	−0.007 (3)
C25	0.046 (3)	0.070 (4)	0.063 (3)	0.003 (3)	0.014 (3)	0.011 (3)
C26	0.072 (4)	0.058 (3)	0.057 (3)	0.005 (3)	0.011 (3)	0.008 (3)
C27	0.056 (4)	0.079 (4)	0.080 (4)	−0.002 (3)	0.002 (3)	0.004 (4)
C28	0.166 (8)	0.062 (4)	0.088 (4)	−0.022 (4)	0.077 (5)	−0.009 (3)
C29	0.082 (4)	0.052 (4)	0.058 (3)	0.002 (3)	0.019 (3)	0.004 (3)
C30	0.079 (5)	0.059 (4)	0.076 (4)	0.003 (3)	0.028 (3)	0.010 (3)
Cd1	0.0507 (3)	0.0500 (3)	0.0565 (2)	−0.00004 (18)	0.01726 (19)	0.00188 (17)
N1	0.067 (3)	0.054 (3)	0.066 (3)	−0.004 (2)	0.029 (2)	−0.008 (2)
N2	0.106 (4)	0.050 (3)	0.094 (3)	−0.017 (3)	0.045 (3)	−0.014 (3)
N3	0.048 (3)	0.058 (3)	0.059 (2)	0.004 (2)	0.017 (2)	0.009 (2)
N4	0.053 (3)	0.071 (3)	0.074 (3)	−0.003 (2)	0.023 (2)	0.005 (2)
O1	0.0491 (18)	0.123 (3)	0.0752 (18)	0.0087 (18)	0.0131 (15)	0.0229 (18)
O2	0.0491 (18)	0.123 (3)	0.0752 (18)	0.0087 (18)	0.0131 (15)	0.0229 (18)
O3	0.125 (3)	0.0522 (18)	0.0756 (17)	0.0066 (17)	0.0499 (18)	0.0068 (14)
O4	0.125 (3)	0.0522 (18)	0.0756 (17)	0.0066 (17)	0.0499 (18)	0.0068 (14)

### Geometric parameters (Å, °)

**Table:**

C1—O2	1.236 (6)	C18—C19	1.412 (10)
C1—O1	1.240 (6)	C18—H18	0.9300
C1—C2	1.513 (7)	C19—C20	1.407 (9)
C2—C3	1.501 (8)	C19—C24	1.426 (8)
C2—H2A	0.9700	C20—C21	1.340 (11)
C2—H2B	0.9700	C20—H20	0.9300
C3—C4	1.366 (8)	C21—C22	1.386 (11)
C3—C12	1.425 (7)	C21—H21	0.9300
C4—C5	1.410 (9)	C22—C23	1.350 (10)
C4—H4	0.9300	C22—H22	0.9300
C5—C6	1.362 (7)	C23—C24	1.423 (9)
C5—H5	0.9300	C23—H23	0.9300
C6—C7	1.413 (7)	C25—N3	1.313 (6)
C6—H6	0.9300	C25—N4	1.323 (7)
C7—C8	1.414 (7)	C25—H25	0.9300
C7—C12	1.422 (7)	C26—C27	1.340 (8)
C8—C9	1.354 (8)	C26—N3	1.365 (7)
C8—H8	0.9300	C26—H26	0.9300
C9—C10	1.402 (9)	C27—N4	1.367 (8)
C9—H9	0.9300	C27—H27	0.9300
C10—C11	1.356 (8)	C28—N1	1.315 (7)
C10—H10	0.9300	C28—N2	1.319 (7)
C11—C12	1.402 (7)	C28—H28	0.9300
C11—H11	0.9300	C29—N1	1.352 (6)
C13—O3	1.230 (6)	C29—C30	1.355 (7)
C13—O4	1.252 (6)	C29—H29	0.9300
C13—C14	1.517 (7)	C30—N2	1.339 (7)
C14—C15	1.494 (8)	C30—H30	0.9300
C14—H14A	0.9700	Cd1—N3	2.244 (4)
C14—H14B	0.9700	Cd1—N1	2.259 (4)
C15—C16	1.367 (9)	Cd1—O3	2.306 (3)
C15—C24	1.415 (9)	Cd1—O1	2.384 (4)
C16—C17	1.398 (10)	Cd1—O2	2.384 (4)
C16—H16	0.9300	Cd1—O4	2.473 (4)
C17—C18	1.353 (11)	N2—H2	0.8600
C17—H17	0.9300	N4—H4A	0.8600
			
O2—C1—O1	120.6 (5)	C21—C20—H20	119.6
O2—C1—C2	122.0 (5)	C19—C20—H20	119.6
O1—C1—C2	117.4 (4)	C20—C21—C22	119.8 (9)
C3—C2—C1	115.3 (4)	C20—C21—H21	120.1
C3—C2—H2A	108.4	C22—C21—H21	120.1
C1—C2—H2A	108.4	C23—C22—C21	121.7 (8)
C3—C2—H2B	108.4	C23—C22—H22	119.1
C1—C2—H2B	108.4	C21—C22—H22	119.1
H2A—C2—H2B	107.5	C22—C23—C24	121.3 (7)
C4—C3—C12	118.8 (5)	C22—C23—H23	119.4
C4—C3—C2	120.5 (5)	C24—C23—H23	119.4
C12—C3—C2	120.6 (5)	C15—C24—C23	123.8 (6)
C3—C4—C5	121.5 (5)	C15—C24—C19	120.4 (6)
C3—C4—H4	119.3	C23—C24—C19	115.8 (7)
C5—C4—H4	119.3	N3—C25—N4	112.0 (5)
C6—C5—C4	120.5 (5)	N3—C25—H25	124.0
C6—C5—H5	119.7	N4—C25—H25	124.0
C4—C5—H5	119.7	C27—C26—N3	110.7 (5)
C5—C6—C7	120.3 (5)	C27—C26—H26	124.7
C5—C6—H6	119.8	N3—C26—H26	124.7
C7—C6—H6	119.8	C26—C27—N4	105.4 (6)
C6—C7—C8	121.1 (5)	C26—C27—H27	127.3
C6—C7—C12	119.0 (5)	N4—C27—H27	127.3
C8—C7—C12	119.9 (5)	N1—C28—N2	112.0 (5)
C9—C8—C7	120.5 (6)	N1—C28—H28	124.0
C9—C8—H8	119.8	N2—C28—H28	124.0
C7—C8—H8	119.8	N1—C29—C30	110.4 (5)
C8—C9—C10	119.9 (6)	N1—C29—H29	124.8
C8—C9—H9	120.1	C30—C29—H29	124.8
C10—C9—H9	120.1	N2—C30—C29	105.4 (5)
C11—C10—C9	120.7 (6)	N2—C30—H30	127.3
C11—C10—H10	119.7	C29—C30—H30	127.3
C9—C10—H10	119.7	N3—Cd1—N1	104.92 (16)
C10—C11—C12	121.8 (6)	N3—Cd1—O3	113.55 (17)
C10—C11—H11	119.1	N1—Cd1—O3	86.72 (14)
C12—C11—H11	119.1	N3—Cd1—O1	85.25 (14)
C11—C12—C7	117.2 (5)	N1—Cd1—O1	110.02 (16)
C11—C12—C3	123.0 (5)	O3—Cd1—O1	151.34 (16)
C7—C12—C3	119.8 (5)	N3—Cd1—O2	138.65 (14)
O3—C13—O4	121.1 (5)	N1—Cd1—O2	87.71 (17)
O3—C13—C14	117.9 (5)	O3—Cd1—O2	106.24 (15)
O4—C13—C14	121.0 (5)	O1—Cd1—O2	53.62 (13)
C15—C14—C13	117.6 (4)	N3—Cd1—O4	100.77 (15)
C15—C14—H14A	107.9	N1—Cd1—O4	139.07 (13)
C13—C14—H14A	107.9	O3—Cd1—O4	53.65 (12)
C15—C14—H14B	107.9	O1—Cd1—O4	103.32 (14)
C13—C14—H14B	107.9	O2—Cd1—O4	93.62 (16)
H14A—C14—H14B	107.2	C28—N1—C29	104.2 (5)
C16—C15—C24	118.3 (6)	C28—N1—Cd1	125.4 (4)
C16—C15—C14	120.4 (7)	C29—N1—Cd1	130.3 (3)
C24—C15—C14	121.3 (6)	C28—N2—C30	107.8 (5)
C15—C16—C17	121.9 (8)	C28—N2—H2	126.1
C15—C16—H16	119.1	C30—N2—H2	126.1
C17—C16—H16	119.1	C25—N3—C26	104.5 (5)
C18—C17—C16	120.7 (8)	C25—N3—Cd1	123.6 (4)
C18—C17—H17	119.6	C26—N3—Cd1	131.7 (3)
C16—C17—H17	119.6	C25—N4—C27	107.4 (5)
C17—C18—C19	120.5 (8)	C25—N4—H4A	126.3
C17—C18—H18	119.7	C27—N4—H4A	126.3
C19—C18—H18	119.7	C1—O1—Cd1	92.6 (3)
C20—C19—C18	121.2 (7)	C1—O2—Cd1	92.7 (3)
C20—C19—C24	120.6 (7)	C13—O3—Cd1	96.7 (3)
C18—C19—C24	118.2 (8)	C13—O4—Cd1	88.2 (3)
C21—C20—C19	120.8 (8)		

### Hydrogen-bond geometry (Å, °)

**Table:**

*D*—H···*A*	*D*—H	H···*A*	*D*···*A*	*D*—H···*A*
N2—H2···O4^i^	0.86	1.92	2.735 (6)	159
N4—H4A···O2^ii^	0.86	1.95	2.772 (6)	159

Symmetry codes: (i) −*x*+1/2, *y*+1/2, −*z*+1/2; (ii) *x*−1, *y*, *z*.
